# Acute Simian Immunodeficiency Virus Infection Triggers Early and Transient Interleukin-7 Production in the Gut, Leading to Enhanced Local Chemokine Expression and Intestinal Immune Cell Homing

**DOI:** 10.3389/fimmu.2017.00588

**Published:** 2017-05-19

**Authors:** Rosalie Ponte, Magali Rancez, Suzanne Figueiredo-Morgado, Jacques Dutrieux, Véronique Fabre-Mersseman, Bénédicte Charmeteau-de-Muylder, Thomas Guilbert, Jean-Pierre Routy, Rémi Cheynier, Anne Couëdel-Courteille

**Affiliations:** ^1^Cytokines and Viral Infections, Immunology Infection and Inflammation Department, Institut Cochin, INSERM, U1016, Paris, France; ^2^CNRS, UMR8104, Paris, France; ^3^Université Paris Descartes, Sorbonne Paris Cité, Paris, France; ^4^McGill University Health Centre, Montréal, QC, Canada; ^5^Université Paris Diderot, Paris, France

**Keywords:** acute HIV/simian immunodeficiency virus infection, non-human primates, Chinese rhesus macaque, interleukin-7, intestinal mucosa, macrophage, chemokine, homing

## Abstract

The intestinal barrier, one of the first targets of HIV/simian immunodeficiency virus (SIV) is subjected to major physiological changes during acute infection. Having previously shown that pharmaceutical injection of interleukin-7 (IL-7) triggers chemokine expression in many organs leading to massive T-cell homing, in particular to the intestine, we here explored mucosal IL-7 expression as part of the cytokine storm occurring during the acute phase of SIV infection in rhesus macaques. Quantifying both mRNA and protein in tissues, we demonstrated a transient increase of IL-7 expression in the small intestine of SIV-infected rhesus macaques, starting with local detection of the virus by day 3 of infection. We also observed increased transcription levels of several chemokines in the small intestine. In infected macaques, ileal IL-7 expression correlated with the transcription of four of these chemokines. Among these chemokines, the macrophage and/or T-cell attractant chemokines CCL4, CCL25, and CCL28 also demonstrated increased transcription in uninfected IL-7-treated monkeys. Through immunohistofluorescence staining and image analysis, we observed increased CD8^+^ T-cell numbers and stable CD4^+^ T-cell counts in the infected lamina propria (LP) during hyperacute infection. Concomitantly, circulating CCR9^+^beta7^+^ CD4^+^ and CD8^+^ T-cells dropped during acute infection, suggesting augmented intestinal homing of gut-imprinted T-cells. Finally, CD4^+^ macrophages transiently decreased in the submucosa and concentrated in the LP during the first days of infection. Overall, our study identifies IL-7 as a danger signal in the small intestine of Chinese rhesus macaques in response to acute SIV infection. Through stimulation of local chemokine expressions, this overexpression of IL-7 triggers immune cell recruitment to the gut. These findings suggest a role for IL-7 in the initiation of early mucosal immune responses to SIV and HIV infections. However, IL-7 triggered CD4^+^ T-cells and macrophages localization at viral replication sites could also participate to viral spread and establishment of viral reservoirs.

## Introduction

Interleukin-7 (IL-7) plays a central role in T-cell development and homeostasis (1, 2). IL-7 has been shown to be constitutively produced by different stromal cells from lymphoid [thymus, bone marrow, and lymph nodes (LNs)] and non-lymphoid (intestine, skin, and liver) organs; its production is unaffected by most extrinsic stimuli. It was thus thought that plasma IL-7 levels are mostly regulated by consumption through binding to its receptor rather than by the amount of IL-7 expression (1, 2). However, in mice, commensal bacteria-driven interferon (IFN)-γ expression by lymphocytes, which regulates basal IL-7 production by epithelial cells, may participate in both T-cell and epithelial cell homeostasis (3, 4). Several studies showed that IL-7 could be regulated in response to immune modulators such as TGF-β, TNF-α, and IFNs in epithelial and endothelial cell lines or in primary stromal cells *in vitro* (5–8). Moreover, IL-7 is increased during inflammation *in vivo* in tagged-IL-7 mice (9, 10). In humans, it has also been reported that IL-7 is locally elevated in the joints of patients with rheumatoid arthritis (11), as well as in the plasma of non-lymphopenic, acutely HCV-infected individuals (12). In lymphopenia, lymphatic endothelial cells produce IL-7 that increases plasma levels (13–15). This production of IL-7, which contributes to LN microenvironment remodeling (14, 15), could play a role in inducing an efficient immune response (15, 16).

During chronic HIV/simian immunodeficiency virus (SIV) infection, plasma IL-7 levels increase with the establishment of lymphopenia, patients with lower than 200 CD4^+^ cells/mL presenting with the highest IL-7 plasma levels (17, 18). In AIDS patients with deep lymphopenia, overexpression of IL-7 by dendritic-like cells or macrophages was evidenced in LNs (17). High plasma IL-7 levels are suspected to play a role in chronic immune activation that characterizes chronically HIV-infected patients (19). Similarly, TLR-dependent IL-7 expression by the liver participates in systemic immune activation during chronic HCV infection (20). IL-7 participates to the massive cytokine storm observed during acute HIV infections (21) and, among other cytokines, was associated with higher viral load and quicker disease progression (22). IL-7 overexpression was also observed during acute *Citrobacter rodentium* infection in mice (23). Produced by colonic epithelial cells, IL-7 is crucial for initiating the very early phase of the immune response (23).

In both steady-state and inflammatory conditions, immune cell homing to and within the intestinal mucosa is regulated by various homeostatic or inflammatory chemokines (24). CCL20 and CCL25, which are produced by epithelial cells in the small intestine, respectively, participate in the steady-state maintenance of CCR6^+^ and CCR9^+^ lymphocyte traffic into organized lymphoid structures (25–27). Indeed, together with α4β7 integrins, CCR9 expression triggers specific T-cell homing into the small bowel (25). Similarly, CCL25, CCL28, and α4 play an important role in the extravasation of IgA-producing plasma cells to the small intestine lamina propria (LP) (28). CCL19 and CCL21, which are expressed by endothelial and stromal cells, attract CCR7^+^ cells into lymphoid aggregates. CXCL12 participates in the localization of plasma cells and T-cells into both the follicles and into the LP (24, 29). During an inflammatory response, immune cell homing to the gut requires CCL2, CCL3, CCL4, and CCL5, as well as CXCL10, which are mostly expressed by epithelial cells (24). Moreover, inflammatory cytokines such as TNF-α and IL-1α induce chemokine expression in the small intestine and in the colon (27).

Regardless of the infection route or animal model utilized, the gastrointestinal tract is one of the first tissues targeted during pathogenic SIV infection, resulting in the rapid impairment of mucosal homeostasis (30, 31). Among the several immune cell subsets shown as depleted during the acute phase of SIV infection, gut LP CD4^+^ T-cells were the most often described, this process being considered as major determinant for disease progression. However, severe CD4^+^ T-cell depletion in the gut has also been observed in non-pathogenic SIV infection (32, 33). Moreover, Allers et al. recently reported that the absolute number of mucosal CD4^+^ T-cells in acutely infected HIV individuals was not different from those of healthy individuals (34). Since the early events occurring after HIV infection play a determining role in clinical outcome, it is important to better understand how HIV affects immune cells during this phase.

Upon IL-7 therapy, transient lymphopenia was observed in HIV-infected patients (35, 36) as well as in healthy macaques (37). In these trials, IL-7-dependent chemokines expression in organs, together with overexpression of chemokine receptors and α4β7 by circulating T-cells lead to massive T-cell homing into the gut (37, 38) and, eventually, improvement of mucosal abnormalities (39). In the acutely SIV-infected macaque model, Vassena and colleagues evidenced a relative protection of peripheral CD4^+^ T-cell pool in IL-7-treated monkeys and moderate increase in viral loads (40).

We here assessed IL-7 expression and comprehensively studied chemokine networks and immune cell subsets in the gastrointestinal tract of Chinese rhesus macaques during acute SIVmac_251_ infection, with a particular focus on CD4^+^ macrophages and T-cells. We show for the first time that SIV infection triggers rapid IL-7 expression in the small intestine of rhesus macaques. This in turn stimulates local expression of many chemokines involved in immune cell recruitment into and within the intestinal mucosa.

## Materials and Methods

### Monkeys, Injections, and Tissue Sampling

Thirty-one adult rhesus macaques (*Macaca mulatta*) of Chinese origin were included in this study.

Four healthy macaques received a single subcutaneous injection of recombinant glycosylated simian IL-7 (R-sIL-7gly; 80 µg/kg body weight); the IL-7 was provided by Cytheris SA; Issy les Moulineaux; France. The macaques were euthanized at 12 h (*n* = 1), 1 day (*n* = 2), or 7 (*n* = 1) days after IL-7-treatment.

Five uninfected healthy macaques (#1–#5) were euthanized and served as controls.

Twenty-two macaques were infected by intravenous injection of 50 AID_50_ of the pathogenic SIVmac_251_ isolate (kindly provided by Dr. Anne-Marie Aubertin; INSERM U544, Strasbourg, France). All macaques were productively infected, as demonstrated by positive plasma SIV viral load by days 3–7.

Nine SIV-infected macaques were euthanized at day 3 (*n* = 3; #6, #7, and #8), 7 (*n* = 2; #9 and #10), 10 (*n* = 2; #11 and #12), or 14 (*n* = 2; #13 and #14). One SIV-infected macaque was euthanized 1-year post-infection because of AIDS-related symptoms (weight loss, cachexia, and prostration). Samples from two other animals euthanized at day 65 were also included in some experiments (samples generously provided by Dr Michaela Müller-Trutwin—Institut Pasteur, Paris).

Organs were collected at necropsy and treated immediately for future analyses. Samples were either frozen at −80°C for future DNA and RNA extraction or snap frozen in liquid nitrogen in optimum cutting temperature (O.C.T.) compound (Labonord, Templemars, France) and preserved at −80°C.

The 10 remaining SIV-infected macaques were longitudinally followed during the acute phase of SIV infection. Blood was sampled every 3–4 days for 3 weeks (10 mL was collected on EDTA).

### IL-7 Quantification

Frozen intestinal tissue samples (20–30 mg) were directly lysed in 500 µL of ice cold Tris Lysis Buffer (Meso Scale Discovery, Rockville, MD, USA) supplemented with protease inhibitors (4× of complete EDTA-free protease inhibitor cocktail, Roche Diagnostics, Meylan, France). Sample grinding consisted of two rounds of 2 min in the TissueLyser II system at 30 Hz with a 10-min incubation on ice in between the cycles. After 30 min of incubation on ice, lysed samples were centrifuged at high speed for 10 min at 4°C to remove cell debris and aliquots were frozen at −80°C. Total protein concentration was quantified using the Pierce Bicinchoninic Acid Assay (BCA Protein Assay Kit, Thermo Fisher Scientific, Villebon sur Yvette, France). IL-7 quantification was performed using the Quantikine Immunoassay IL-7 HS (R&D Systems, Lille, France), according to the manufacturer’s instructions.

### Real-time PCR Quantifications

Total mRNA was extracted from homogenized tissue (20–30 mg) using the RNeasy kit (Qiagen, Les Ulis, France), including 1 step of DNase treatment (RNase-Free DNase Set, Qiagen). RNA was reverse transcribed using the QuantiTect Reverse Transcription Kit (Qiagen). Specific cDNAs were PCR-amplified using “outer” 3′/5′ primer pairs by 15 min of denaturation at 95°C, followed by 22 cycles of 30 s at 95°C, 30 s at 60°C, and 3 min at 72°C. Each target was coamplified together with HPRT sequences; HPRT was used as a housekeeping gene. Target mRNA and HPRT were quantified within each of the PCR products in LightCycler^®^ experiments performed on 1/280th of the PCR products; “inner” 3′/5′ primer pairs and the LightCycler^®^480 SYBR Green I Master Mix (Roche Diagnostics, Meylan, France) were used. The PCR cycling program consisted of 10 min of initial denaturation at 95°C, 40 cycles of 10 s at 95°C, 6 s at 64°C, and 15 s at 72°C. Fluorescence measurements were performed at the end of the elongation steps. Plasmids containing one copy of both the HPRT gene and of the target amplicons were used to generate standard curves. IL-7, chemokines, and HPRT mRNA quantifications were performed in independent experiments using the same first-round serial dilution standard curve. Quantifications were made in triplicate for all samples studied; three to five samples were used for each tissue from each macaque. The target mRNA concentration was normalized to HPRT mRNA in each sample. The results were expressed as the absolute number of target mRNA copies per HPRT mRNA copy. HPRT-, IL-7-, and chemokine-specific primers are described in Table S1 in Supplementary Material.

### Viral RNA and DNA Quantifications

A quantification method similar to the one described above for IL-7 and chemokine quantification was used to quantify total SIV RNA; Gag-specific primers were used (Table S2 in Supplementary Material).

For SIV DNA quantifications, tissues were lysed in Tween-20 (0.05%), Nonidet P-40 (0.05%), and proteinase K (100 µg/mL) for 30 min at 56°C, followed by 15 min at 98°C. SIV DNA was directly quantified from the lysate using Gag-specific primers (Table S2 in Supplementary Material). Gag sequences were amplified together with the rhesus macaque CD3γ chain in triplicate using the “outer” 3′/5′ primer pairs and the same conditions as described earlier. SIV Gag and CD3γ were then quantified using the “inner” 3′/5′ primer pairs (Table S2 in Supplementary Material) and a plasmid containing one copy of both the CD3γ and Gag amplicons. The results were expressed as the absolute number of SIV copies per 10^6^ cells.

### Flow Cytometry Analysis

Peripheral blood cell phenotype was analyzed from frozen whole blood by flow cytometry using a BD FACS CANTO II (BD Biosciences, Le Pont de Claix, France) as described (41). The fluorochrome-conjugated antibodies used for polycromatic flow cytometric analysis are listed in Table S3 in Supplementary Material.

### Immunohistofluorescence Staining

Ileum cryosections 7 μm in thickness were placed on SuperFrost^®^ Plus slides (Thermo Scientific Menzel, Braunschweig, Germany), fixed for 10 min at 4°C in acetone/methanol (50/50), rinsed in PBS, and blocked with 5% BSA/2% normal goat serum in PBS at room temperature (RT) for 30 min. Sections were incubated overnight with primary antibodies in a 4°C humid chamber. Sections were rinsed in 0.5% Tween20/PBS, incubated at RT in the dark for 30 min with secondary antibodies, and rinsed in 0.5% Tween20/PBS. Finally, sections were washed in PBS alone, counterstained with 4,6-diamidino-2-phenylindol (DAPI; Molecular Probes, Cergy Pontoise, France), and mounted in Fluoromount-G medium (Southern Biotechnology, Birmingham, AL, USA). Cell quantifications in tissue were performed on large (25 mm^2^) images reconstructions of at least two samples per macaque acquired with 20× oil objective (Leica Microsystems Gmbh, Wetzlar, Germany) using the Yokogawa CSU X1 Spinning Disk (Yokogawa, Tokyo, Japan) coupled with a DMI6000B Leica microscope with MetaMorph 7.7 software (Molecular Devices, Sunnyvale, CA, USA), using the Scan-Slide option (10% overlap). To identify IL-7 in the infected mucosa, immunostaining experiments were performed using polyclonal antibodies directed against IL-7, cytokeratin, and ezrin (polyclonal antiserum obtained from P. Mangeat; CNRS UMR 5539, Montpellier, France). Acquisitions were performed with 20× objective on a DMI6000B Leica microscope with MetaMorph 7.7 software. The antibodies used for immunohistofluorescence labeling are listed in Table S4 in Supplementary Material.

### Quantitative Image Analysis

Analyses were performed using the ImageJ software.^1^ In order to accurately quantify nuclei (DAPI), CD3^+^ cells (Alexa Fluor^®^ 546), or CD4^+^, or CD8^+^ cells (Alexa Fluor^®^ 488), images were acquired over the entire thickness of the sample with 13–20 *z*-stack images and the best focuses were chosen. In order to count immune cells in large areas, the LP, submucosa (SM), and regions containing epithelial cells (E) or lymphoid follicles (LF) were manually defined. Pixels corresponding to E + LF zones were excluded from the process. These excluded zones were replicated on all treated images. After a denoising process, a manual threshold was applied on CD3^+^, CD4^+^, and CD8^+^ stains to identify immune cell surfaces. CD3^−^CD4^+^ and CD3^−^CD8^+^ stained surfaces, as well as CD3^+^CD4^+^ and CD3^+^CD8^+^ costained surfaces, were quantified. Results were presented as total single-stained or costained surfaces/total surface of LP or SM. At least 1.5 mm^2^ of LP and 1.4 mm^2^ of SM surfaces were analyzed per macaque.

### Statistical Analysis

The non-parametric Spearman rank correlation test was used to investigate the relationship between parameters. The non-parametric Mann–Whitney test was used to compare different groups of macaques, and the non-parametric Wilcoxon rank sum test was used to compare data from the same macaques at different time points before and after SIV infection. All statistical analyses were performed with Real Statistics^2^ add-in to Microsoft Excel software. In two-tailed tests, *p* values of 0.05 or lower were considered significant.

### Ethics Statement

All the animals included in this study were housed, cared for, and handled in BSL2 (uninfected macaques) or BSL3 (SIV-infected macaques) facilities at the Institut Pasteur (Paris, France; accreditation no. A 78-100-3) and IDMIT (“Infectious Disease Models and Innovative Therapies” at the CEA “Commissariat à l’Energie Atomique,” Fontenay-aux-Roses, France; accreditation no. C 92-032-02) NHP facilities, licensed by the French Ministry of Agriculture, in accordance with European guidelines for animal care (European directive 86/609, “Journal Officiel des Communautés Européennes,” L358, December 18, 1986). The CEA is in compliance with Standards for Human Care and Use of Laboratory of the Office for Laboratory Animal Welfare (OLAW, USA) under OLAW Assurance number #A5826-01. This study and the procedures used were approved by the “Comité Régional d’Ethique en Expérimentation Animale Ile-de-France—Paris—Comité 1,” which follows the governance of the “Charte nationale portant sur l’expérimentation animale” according to the EU Directive 2010/63 on the protection of animals used for scientific purposes (notifications no. 2011-0001 and 2012-0003). All experimental procedures were carried out in strict compliance with European guidelines for NHP care (European Directive 86/609 then; as of January 2013, EU Directive N 2010/63/EU) for protection of animals used for experimentation and other scientific purposes and the Weatherall report. Food and water were provided *ad libitum*. Temperature was maintained constant (22 ± 2°C). Air was renewed at least 20 times per hour. Fluorescent light was provided with a 12:12 h light–dark cycle. All efforts were made to minimize suffering, including improving housing conditions and providing enrichment opportunities (e.g., provision of monkey biscuits supplemented with fresh fruit and constant water access, objects to manipulate, interaction with caregivers and research staff). Animals were kept in cages in compliance with European regulations in terms of floor surface per animal. All manipulations (injections, blood draws, and euthanasia) were performed under anesthesia (tiletamine/zolasepam; 4.6 mg/kg). Euthanasia was performed by intravenous injection of pentobarbital (10 mL). Permanent veterinarians managed the animal facilities and were responsible for the well-being of the experimental animals. The animal facilities were run by teams of animal caretakers under the supervision of animal technicians. According to European and French regulations, animal caretakers have received a specific training in laboratory animal science. Technicians who were doing experiments on live animals had received the appropriate training. Biohazards were handled in BSL2 and BSL3 facilities according to French legislation.

## Results

### Local IL-7 Overexpression Coincides with SIV Infection in the Small Intestine

**Figure 1:**
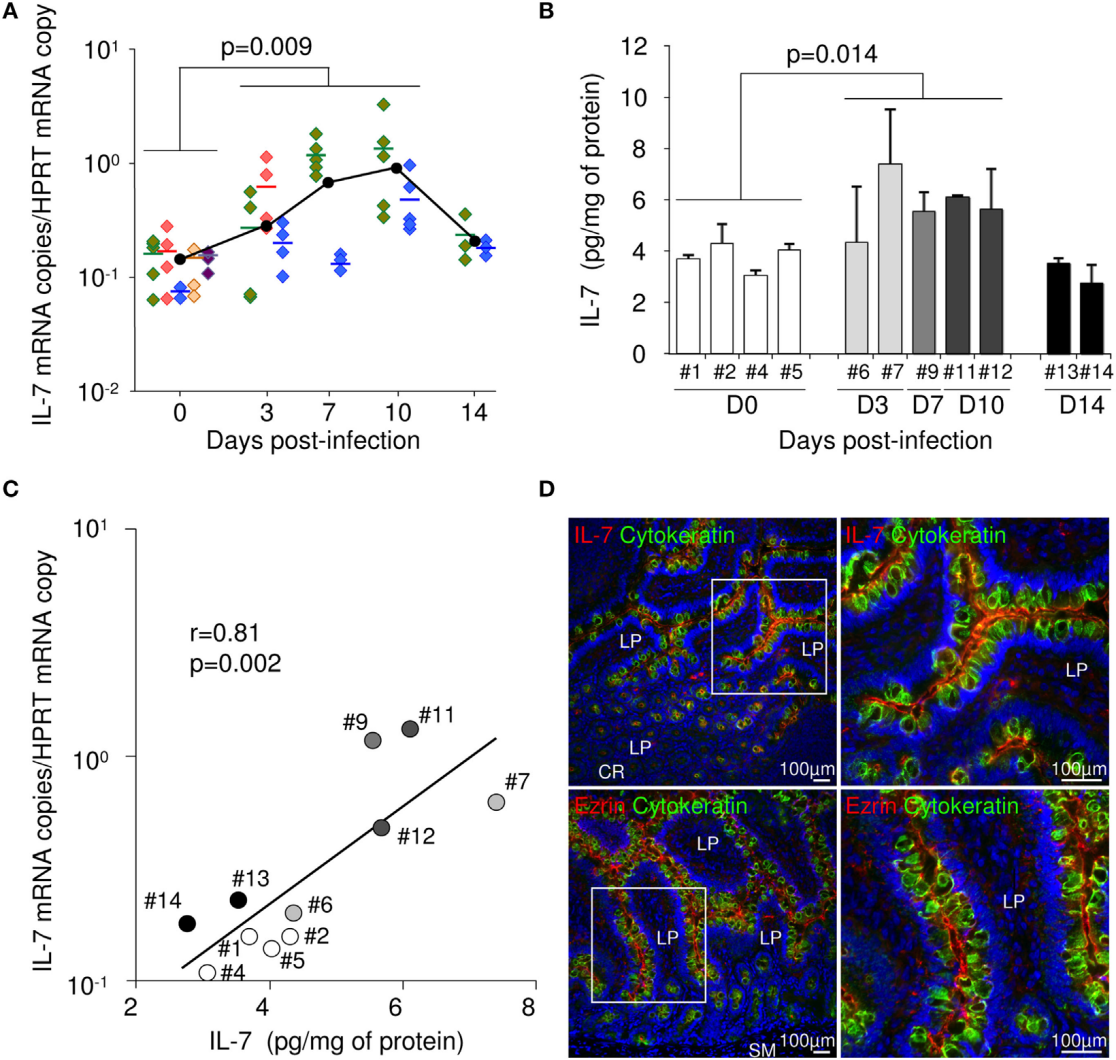
**Interleukin (IL)-7 expression in the ileum of rhesus macaques during acute simian immunodeficiency virus (SIV) infection**. **(A)** IL-7 mRNA concentration in the ileum was quantified by RT-qPCR in healthy macaques (#1 green, #2 red, #3 blue, #4 brown, and #5 purple) and macaques sacrificed on days 3 (#6 green, #7 red, and #8 blue), 7 (#9 green and #10 blue), 10 (#11 green and #12 blue), or 14 (#13 green and #14 blue) following SIVmac_251_ infection (IL-7 mRNA copies/HPRT mRNA copy). At each time point, each color represents one animal. For each macaque, each symbol represents an individual sample of ileal tissue, means are shown as horizontal bars. Black dots represent the median of the macaques sampled at each time point. Statistical significance between infected monkeys (days 3, 7, and 10) and healthy monkeys is shown; Mann–Whitney one-tail *U* test. **(B)** IL-7 concentration was assessed by ELISA in ileal tissue sampled from both healthy macaques and macaques sacrificed at days 3, 7, 10, or 14 following SIVmac_251_ infection. Bars and error bars represent means and SDs, respectively. Statistical significance between pre- and post-infection samples is shown; Mann–Whitney one-tail *U* test. **(C)** Correlation between IL-7 concentration and IL-7 mRNA levels in ileal tissue sampled from healthy and SIV-infected macaques. Each point represents one animal. White circles represent uninfected controls; light gray, medium gray, dark gray, and black circles represent infected animals sampled at days 3, 7, 10, or 14, respectively. Regression line, Spearman’s rank correlation value, and associated probability are shown. **(D)** Immunohistofluorescent labeling for IL-7 (top panels; red) or Ezrin (bottom panels; red) and cytokeratin (all panels; green) on cryosections of ileal tissue sampled at day 10 pi. Right panels represent higher magnifications of squared areas on left panels. Nuclei are labeled in blue with DAPI. LP, lamina propria; SM, submucosa; CR, crypt.

Intestinal tissue and LNs were sampled from five healthy and nine SIV-infected Chinese rhesus macaques sacrificed at days 3 (*n* = 3), 7 (*n* = 2), 10 (*n* = 2), or 14 (*n* = 2) following intravenous infection with 50 AID_50_ of SIVmac_251_. In these tissues, we first evaluated IL-7 expression by measuring mRNA levels using a highly sensitive qRT-PCR assay. Interestingly, IL-7 transcription significantly rose by day 3 in the ileum. Maximal IL-7 transcription was observed at day 7–10 post-infection (pi) (*p* = 0.009; Figure 1A). IL-7 expression returned close to baseline values at day 14. IL-7 mRNA levels were also increased by day 3 in the duodenum and the jejunum, slightly increased at day 10 in the large bowel and remained stable in the LNs, suggesting that the increase in IL-7 transcription was mostly confined to the small bowel in acutely SIV-infected macaques (data not shown). Quantification of IL-7 protein in the ileal tissue also demonstrated that IL-7 expression was higher in macaques sampled throughout acute infection than in uninfected monkeys. Mean local IL-7 concentration was 5.8 pg/mg of total protein from days 3 to 10, as compared to 3.7 pg/mg in uninfected controls (*p* = 0.014; Figure 1B). Moreover, in the ileum, IL-7 mRNA levels directly correlated with IL-7 protein concentration (*r* = 0.81; *p* = 0.002; Figure 1C), suggesting that increased transcription translated in increased protein production. Immunohistochemical staining confirmed epithelial cells as a possible source of intestinal IL-7. Indeed, similar to ezrin staining, IL-7 staining mostly localized at the apical pole of the epithelial cells as demonstrated by cytokeratin staining (Figure 1D).

In order to investigate whether intestinal IL-7 production could be initiated by local infection, we quantified SIV DNA and RNA in tissues from SIV-infected monkeys. At early time points, higher levels of SIV replication were observed in the ileum compared to any other organ, including LNs, with viral DNA being detectable by day 3 in all tested ileal samples (Table 1). Large amounts of SIV DNA were observed in most organs sampled at day 10 or 14 post-infection (pi), reaching 10^3^ genomes per million cells in mesenteric LNs, in the ileum, and in the large intestine. In the ileum, SIV DNA load correlates with IL-7 expression (*r* = 0.54, *p* = 0.047; Spearman’s rank correlation).

**Table 1:** **Quantification of simian immunodeficiency virus (SIV) DNA in tissues sampled from SIV-infected rhesus macaques**.

SIV DNA	Mac #	Axillary lymph node (LN)	Mesenteric LN	Duodenum	Jejunum	Ileum	Asc. colon	Desc. colon	Rectum
Day 3	6	+	−	−	−	+	+	+	++
	7	+	−	−	+	++	+	−	−
	8	+	+	+	++	+++	++	+++	++
Day 7	9	−	−	−	+	++	−	+	+
	10	++	+++	+++	++	+++	++	+++	++
Day 10	11	+++	++++	+++	+++	++++	+	++++	+++
	12	+++	++++	+	−	++++	+++	+++	++++
Day 14	13	++++	++++	++++	++++	++++	++++	++++	++++
	14	++++	++++	++++	++++	++++	++++	++	++++

*−, Undetectable; +: 0 < X ≤ 10 copies/10^6^ cells; ++: 10 < X ≤ 100 copies/10^6^ cells; +++: 100 < X ≤ 1,000 copies/10^6^ cells; ++++: X > 10^3^ copies/10^6^ cells*.

Simian immunodeficiency virus replication, defined by the presence of unspliced viral mRNA, was detectable in most organs sampled at days 10 or 14 (Table S5 in Supplementary Material). Sporadic SIV RNA positivity was observed before day 10.

### Strong Chemokine Responses Characterize Acute SIV Infection in the Small Intestine

**Figure 2:**
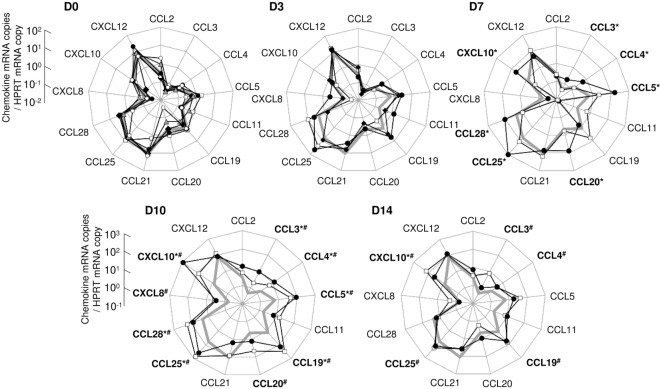
**Assessment of the chemokine expression network in the ileal mucosa of simian immunodeficiency virus-infected macaques**. Thirteen chemokines were quantified by RT-qPCR in ileal tissues sampled at day 0 (D0; macaques #1 open squares, #2 close diamonds, #3 close circles, #4 gray triangles, and #5 open circles), day 3 (D3; #6 open squares, #7 close circles, and #8 close diamonds), day 7 (D7; #9 close circles and #10 open squares), day 10 (D10; #11 close circles and #12 open squares), or day 14 (D14; #13 close circles and #14 open squares) pi (chemokine mRNA copies/HPRT mRNA copy). The bold gray line on days 3, 7, 10, and 14 graphs represents the median of chemokine concentrations of the five healthy macaques (D0). For each macaque, each dot represents the median of three to five independent samples, quantified in triplicate. Statistical analysis was performed individually for each infected macaque, compared to each healthy control. A given chemokine was considered as overexpressed in one infected macaque if significantly different from all healthy controls, tested independently (*p < 0.05 for macaque #9, #11, or #13, as compared to each uninfected monkeys; ^#^*p* < 0.05 for macaque #10, #12, or #14, as compared to each uninfected monkeys; Mann–Whitney two-tailed *U* test).

To further characterize the impact of early infection on the mucosal cytokine/chemokine network, we investigated the expression of 13 chemokines (CCL2, CCL3, CCL4, CCL5, CCL11, CCL19, CCL20, CCL21, CCL25, CCL28, CXCL8, CXCL10, and CXCL12) using real-time quantitative RT-PCR. Tissues were sampled from the gut of macaques sacrificed at days 3, 7, 10, or 14 pi and chemokine levels were compared to values from uninfected controls; the values obtained from these controls served as baseline, i.e., pre-infection values. At baseline, a few chemokines were constitutively expressed in the ileal mucosa (CCL5, CCL19, CCL21, CCL25, CCL28, and CXCL12; Figure 2). Higher chemokine transcription was observed in one infected macaque sacrificed at day 7 (CCL3, CCL4, CCL5, CCL20, CCL25, CCL28, and CXCL10; Macaque #9; Figure 2). At day 10, transcription of CCL3, CCL4, CCL5, CCL19, CCL25, CCL28, and CXCL10 was increased in both animals while CCL20 and CXCL8 were overexpressed in macaque #12 (Figure 2). At day 14, most chemokine levels had returned to close to baseline levels, except for CCL3, CCL4, CCL19, and CCL25 which remained high in one of the two macaque (Macaque #14) and CXCL10 levels which remained elevated in both macaques (Figure 2).

Similarly, several chemokines were overexpressed in the other parts of the small intestine of the macaques sacrificed at day 10 or 14 (CXCL10 in both tissues from macaques #11, #12, #13, and #14; CCL11 and CCL20 in the duodenum of macaques #11 and #12; CCL2, CCL3, CCL5, CCL11, and CXCL8 in the duodenum of macaque #14; CCL5, CCL11, and CCL19 in the jejunum of macaque #14; data not shown).

### IL-7 Drives Chemokine Production in the Small Intestine

**Figure 3:**
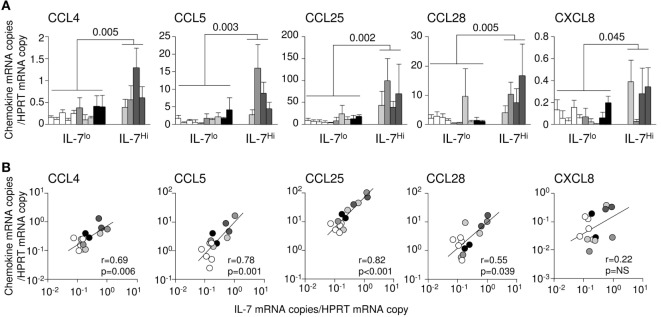
**Interleukin (IL)-7 and chemokine expression in the intestinal tract of simian immunodeficiency virus (SIV)-infected rhesus macaques**. **(A)** Chemokine expression in acutely SIV-infected macaques presenting high (macaques #7, #9, #11, and #12; IL-7^Hi^), or low (uninfected macaques #1, #2, #3, #4, and #5) and infected macaques (#6, #8, #10, #13, and #14; IL-7^Lo^) IL-7 expression in the ileal mucosa. White bars represent uninfected controls; light gray, medium gray, dark gray, and black bars represent infected animals sampled at day 3, 7, 10, and 14, respectively. Bars and error bars represent means and SDs, respectively. **(B)** Correlations between IL-7 and chemokine mRNA expressions in the ileum of healthy and SIV-infected macaques (3–10 days post-SIV infection). Each point represents one animal. White circles represent uninfected controls; light gray, medium gray, dark gray, and black circles represent infected animals sampled at day 3, 7, 10, and 14, respectively. The regression lines, the Spearman’s rank correlation value, and the associated probability are shown.

We had previously demonstrated that IL-7 injection triggers chemokine expression in tissues (37). We therefore examined whether, in infected tissues, endogenous IL-7 could be responsible for the observed local increase of chemokine transcription. Accordingly, classifying the infected monkeys based on ileal IL-7 transcription levels, we established that CCL4, CCL5, CCL25, CCL28, and CXCL8 expressions were significantly increased in the ileum of monkeys demonstrating high IL-7 expression (#7, #9, #11, and #12) as compared to macaques with low IL-7 transcription/expression (#1, #2, #3, #4, #5, #6, #8, #10, #13, and #14; Figure 3A). Moreover, at both tissue sample level (data not shown) and animal level (Figure 3B), IL-7 mRNA levels directly correlated with local mRNA expression of CCL4, CCL5, CCL25, and CCL28 in the ileum (*r* = 0.69, 0.78, 0.82, and 0.55; *p* = 0.006, 0.001, <0.001, and 0.039, respectively); multivariate analysis showed that viral load had no impact on these correlations. Similar correlations were observed in the duodenum (CCL5; *r* = 0.60; *p* = 0.01; Figure S1A in Supplementary Material) and in the jejunum (CCL5, CCL20, and CCL25; *r* = 0.65, 0.70, and 0.78; *p* = 0.01, 0.005, and 0.001, respectively; Figure S1B in Supplementary Material). These data suggest that IL-7 is involved in upregulating the transcription of these chemokine in the infected intestine. By contrast, ileal transcription of CCL2, CCL3, CCL19, and CXCL10 correlated with local SIV DNA (*r* = 0.69, 0.55, 0.76, and 0.61; *p* = 0.01, 0.04, 0.001, and 0.02, respectively; Figure S2 in Supplementary Material) and SIV RNA loads in SIV-infected monkeys (*r* = 0.57, 0.64, 0.66, and 0.65; *p* = 0.03, 0.02, 0.009, and 0.01, respectively; Figure S2 in Supplementary Material), suggesting a direct role for viral infection in increasing the transcription of these chemokines.

**Figure 4:**
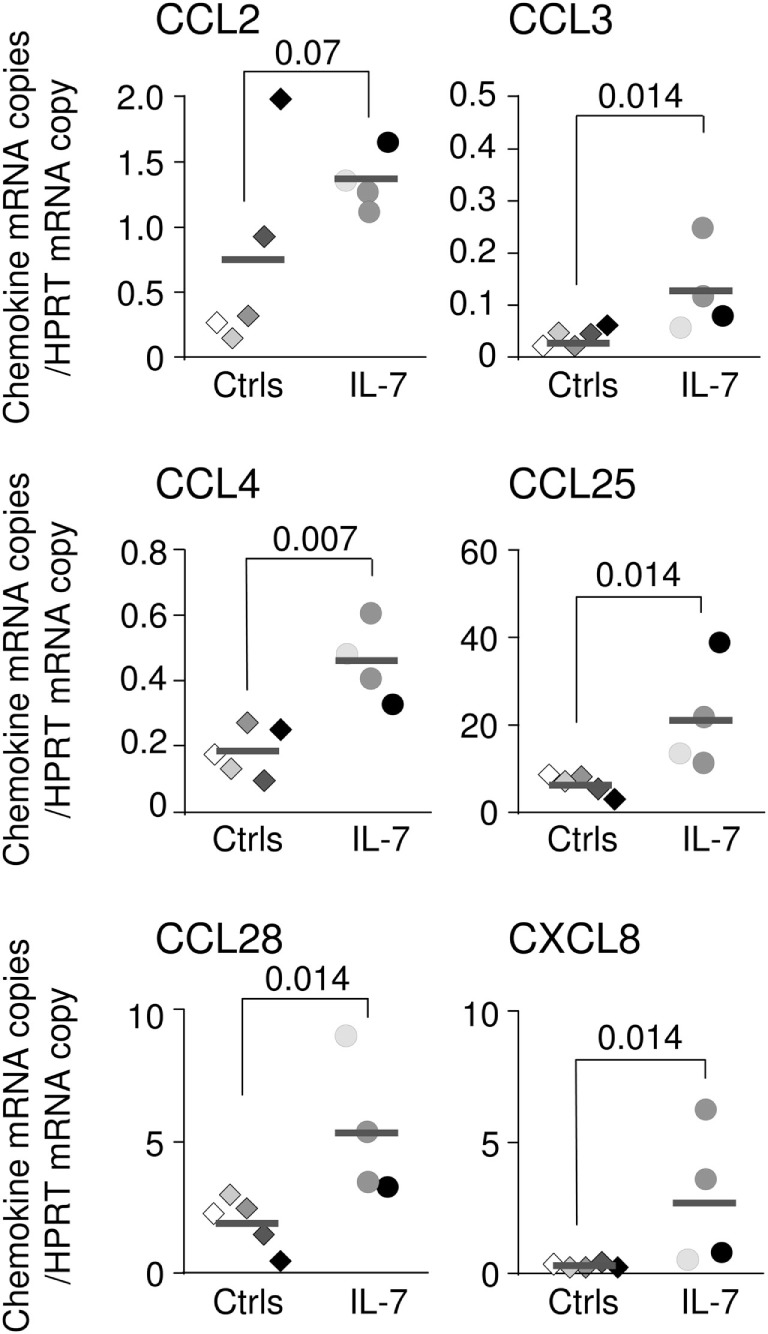
**Chemokine expression in the intestinal tract of interleukin (IL)-7-treated rhesus macaques**. CCL2, CCL3, CCL4, CCL25, CCL28, and CXCL8 mRNA expression in ileal tissues (three to five samples per macaque) sampled from five control rhesus macaques (macaques #1–#5; diamonds) and four healthy animals sacrificed 12 h (light gray circles), 1 day (dark gray circles), or 7 days (black circles) after systemic IL-7 treatment (80 μg/kg of body weight). Medians are shown as horizontal bars. Statistically significant differences between IL-7-treated and untreated monkeys were calculated using Mann–Whitney one-tail *U* test.

To confirm the role of IL-7 in regulating chemokine expression, four uninfected macaques were subcutaneously injected with pharmacological doses of IL-7 (80 μg/kg of body weight) and sacrificed either 12 h, or 1 or 7 days after treatment. Of note, the local IL-7 concentrations measured at day 1 were of the same order of magnitude as those observed in SIV-infected macaques (15–30 pg/mg of total proteins; data not shown). Ileal transcription of CCL3, CCL4, CCL25, CCL28, and CXCL8 was increased in IL-7-treated macaques (*p* < 0.02 as compared to untreated monkeys; Figure 4), a trend being observed for CCL2 (*p* = 0.07; Figure 4). Similarly, increased transcription of CCL4, CCL5, and CCL28 in the duodenum, and of CCL4 and CXCL8 in the jejunum, were observed following systemic IL-7 administration (data not shown).

Collectively, these data suggest that locally produced IL-7 acts as a danger signal that triggers chemokine overexpression in the ileum of acutely SIV-infected Chinese macaques. We hypothesize, therefore, that SIV-induced production of IL-7 expression in the gut may participate in the recruitment of immune cells into the infected mucosa, as observed in IL-7-treated humans and macaques (37, 38).

### Increased CD8^+^ T-Cell Numbers but Stable CD4^+^ T-Cell Numbers in the Ileum during Acute SIV Infection

**Figure 5:**
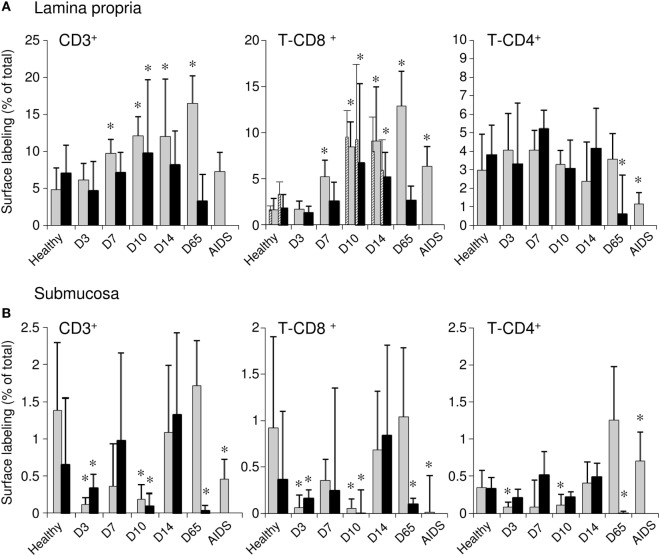
**Changes in the CD4^+^ and CD8^+^ T-cell compartments in the ileum during simian immunodeficiency virus infection**. Ileal tissues sampled from healthy animals as well as from macaques euthanized at 3, 7, 10, 14, or 65 days post-SIV infection (two animals per time point), and from one animal presenting with AIDS-related symptoms, were labeled using anti-CD3 and anti-CD4 antibodies. The proportions of lamina propria [LP; **(A)**] or submucosa [SM; **(B)**] tissues occupied by the CD3^+^, CD3^+^CD4^−^ (T-CD8^+^), and CD3^+^CD4^+^ (T-CD4^+^) labeled cells are represented as medians and SDs for each time point. For each animal, 5–10 independent areas, representing at least 1.5 mm^2^ of LP or 1.4 mm^2^ of SM, were quantified. Hatched bars on the T-CD8^+^ graph [**(A)**, central panel] represent quantifications of CD3^+^CD8^+^ cells (labeled with anti-CD3 and anti-CD8 antibodies) performed on tissues sampled on days 0, 10, or 14 pi. Statistical analysis was performed for each infected macaque, compared individually to each healthy control. A given subset was considered as over/under represented in one infected macaque if significantly different from both healthy controls, tested independently (*p < 0.05 as compared to each uninfected monkeys; Mann–Whitney two-tailed *U* test).

In order to evaluate the possible consequences of local chemokine network changes on immune cell localization in the infected ileum, we quantified immune cell subsets in the ileal mucosa of uninfected macaques or SIV-infected monkeys. Infected macaques were sampled throughout the acute infection, at day 65 pi or at the AIDS disease stage. We observed that despite the relatively homogeneous cellular contents of the LP, as demonstrated by the constant proportion of the mucosal surface that was occupied by nuclei, CD3 surface staining varied considerably in the various LP zones analyzed (Figure S3 in Supplementary Material). In order to minimize the impact of the uneven T-cell distribution observed in both uninfected and infected macaques, immune cell distribution was quantified on entire tissue sections (25 mm^2^ per slide). In SIV-infected macaques, CD3 surface staining increased by day 7 in the ileal LP (9.7, 12.1, 9.8, and 12% of the analyzed surfaces in macaques #9, #11, #12, and #13, respectively, as compared to 4.8 and 7.1% in macaques #1 and #2 sampled at baseline; Figure 5A; left panel). At day 65 pi, CD3^+^ T-cell counts remained high in the ileal LP of one macaque, while it returned to baseline levels in the second macaque. In the monkey with AIDS-related symptoms, 7.2% of the analyzed surface stained for CD3, a value similar to that observed at baseline. The increase in the density of CD3^+^ T-cells observed during the course of the primary infection was almost exclusively due to CD8^+^ T-cells. Indeed, the proportion of the surface area that was stained CD3^+^CD4^−^ increased during acute infection and remained high throughout the study (Figure 5A; central panel), except for the second macaque sampled at day 65 pi. Of note, the values obtained for CD3^+^CD8^+^ cell quantifications of tissues sampled at baseline, days 10 and 14 were very similar to the values obtained for CD3^+^CD4^−^ staining, suggesting that the CD3^+^CD4^−^ cells are in fact CD8^+^ T-cells (hatched bars on Figure 5A; central panel). By contrast, the density of CD4^+^ T-cells (CD3^+^CD4^+^) remained constant until day 65 pi, representing 3–4.2% of the analyzed surfaces at any time point (Figure 5A; right panel). Depletion of ileal LP CD4^+^ T-cells was only observed in both the second macaque sacrificed at D65 pi and the macaque with AIDS-related symptoms (82 and 76% decrease compared to mean values in macaques sampled at baseline, Figure 5A; right panel), consistent with the depletion of gut CD4^+^ T-cells that has been largely described in chronically infected macaques and humans.

Using these same tissue sections, we also analyzed the different cell populations in the submucosa (Figure 5B). Despite low baseline cell concentrations [the median percentage of the tissue surface occupied by nuclei was 31.12% (26.8–63.6)], both CD4^+^ and CD8^+^ T-cell numbers fluctuated over the course of acute infection, with transient declines at days 3 and 10 pi (Figure 5B). At AIDS stage, as well as in the second macaque sampled at D65, CD8^+^ T-cells were almost completely depleted from the submucosa, while CD4^+^ T-cells increased at AIDS stage.

### Increased CD4^+^ Macrophages Concentration in the LP of Acutely Infected Macaques

In the ileal mucosa, T-cells represent only a fraction of the total lymphoid cells. In healthy rhesus macaques, all LP CD8^+^ cells were round and more than 70% coexpressed CD3, marking them as T-cells (Figure S4 in Supplementary Material). By contrast, more than half of mucosal CD4^+^ cells did not coexpress CD3; these cells had the typical shape of antigen-presenting cells (APCs) (Figure S4 in Supplementary Material).

**Figure 6:**
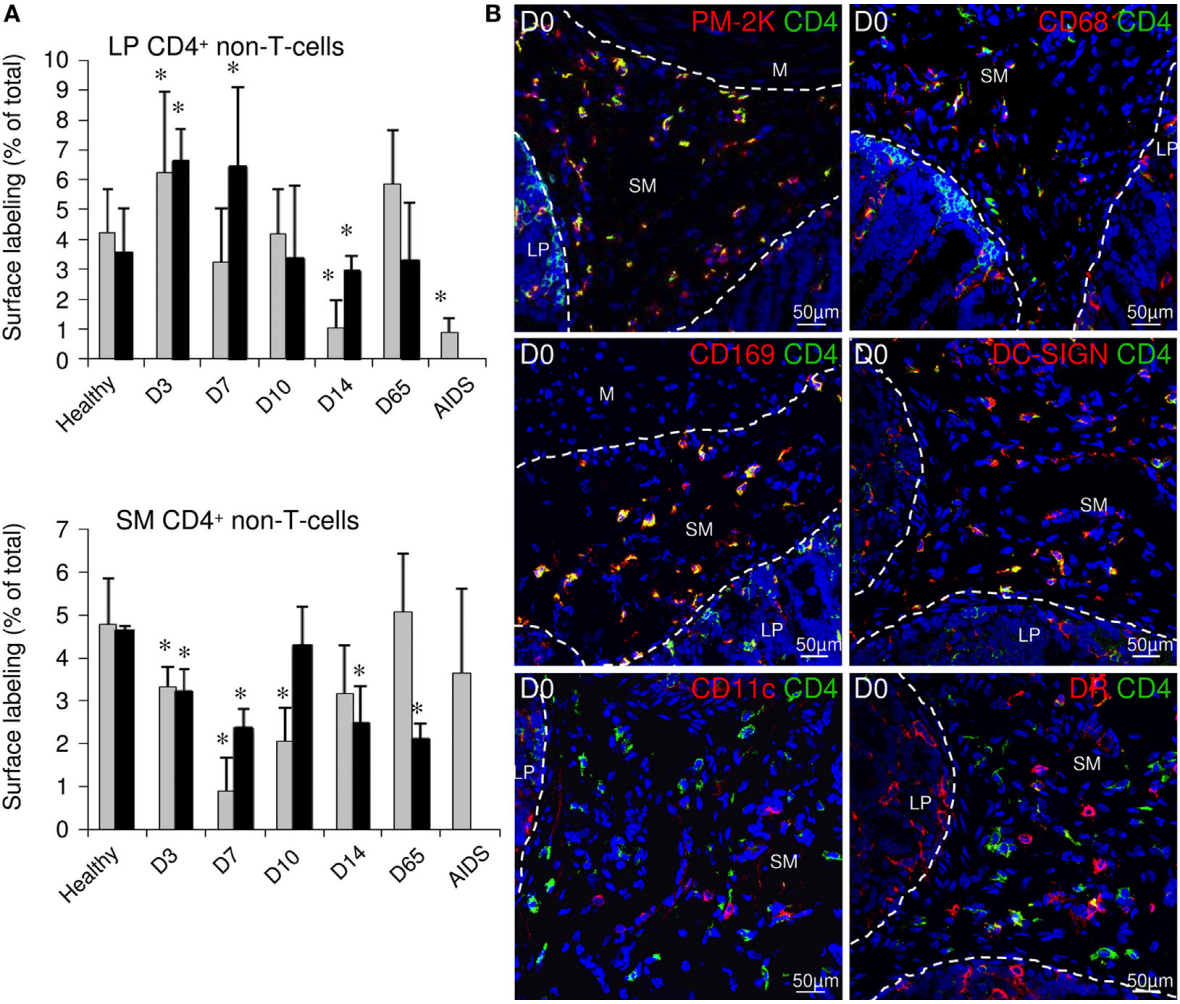
**Quantification of the CD4^+^ non-T-cell subset in the LP and submucosa during simian immunodeficiency virus infection**. **(A)** Ileal tissues sampled from healthy animals as well as from macaques euthanized at 3, 7, 10, 14, or 65 days post-SIV infection (two animals per time point), and from one animal presenting with AIDS-related symptoms, were labeled using anti-CD3 and anti-CD4 antibodies. The proportions of LP (top panel) or SM (bottom panel) tissues occupied by the CD3^−^CD4^+^ labeled cells are represented as medians and SDs for each time point. For each animal, 5–10 independent areas, representing at least 1.5 mm^2^ of LP or 1.4 mm^2^ of SM, were quantified. Statistical analysis was performed for each infected macaque, compared individually to each healthy control. CD4^+^ non-T-cell subset was considered as over/under represented in one infected macaque if significantly different from both healthy controls, tested independently (*p < 0.05 as compared to each uninfected monkeys; Mann–Whitney two-tailed *U* test). **(B)** Representative examples of the ileum LP and submucosa of uninfected macaques colabeled with anti-CD4 monoclonal antibody (green) and anti-PM-2K, CD68, CD169/Siglec-1, DC-SIGN, CD11c, or MHC-II MamuLa-DR (DR) monoclonal antibodies (red). Nuclei are labeled in blue with DAPI. LP, lamina propria; SM, submucosa; M, muscular tissue.

Lamina propria tissues were thus also examined for changes in the proportion of CD4-expressing non-T-cells (CD4^+^CD3^−^) during the course of SIV infection. These CD4^+^CD3^−^ cells rapidly expanded/infiltrated the ileal LP during the first 3 days pi (3.7 and 6.5% of tissue surface at day 0 and day 3, respectively; Figure 6A; top panel) and then gradually declined. At day 14 pi, these cells were less abundant in the LP than before infection (2.5% of tissue surface). This effect was only transient in acute infection, as CD4^+^CD3^−^ stained surfaces returned to baseline levels at day 65 pi. Finally, these cells were barely detectable in late stage disease. In the submucosa, CD4^+^ non-T-cells represented more than 90% of the CD4^+^ surface in healthy macaques (data not shown). This population was almost entirely composed of cells with dendrites that expressed PM-2K, CD68, CD169 (Sialoadhesin/Siglec-1), and DC-SIGN, but not CD11c, marking them as macrophages (Figure 6B). Furthermore, these cells expressed low levels of MamuLa-DR, CD83, and CD86 (B7-2) (Figure 6B; data not shown) and did not proliferate as they were Ki67^−^ (data not shown). In the submucosa, the CD4^+^ macrophage subset rapidly declined during the first days of acute infection; thus, the changes in the numbers of this macrophage population mirrored the changes observed in the LP (Figure 6A; bottom panel).

Altogether, our data suggest that cell migration occurred rapidly into and/or within the ileal mucosa during the first days of SIV infection.

### Changes in T-Cell Subsets in the Blood

**Figure 7:**
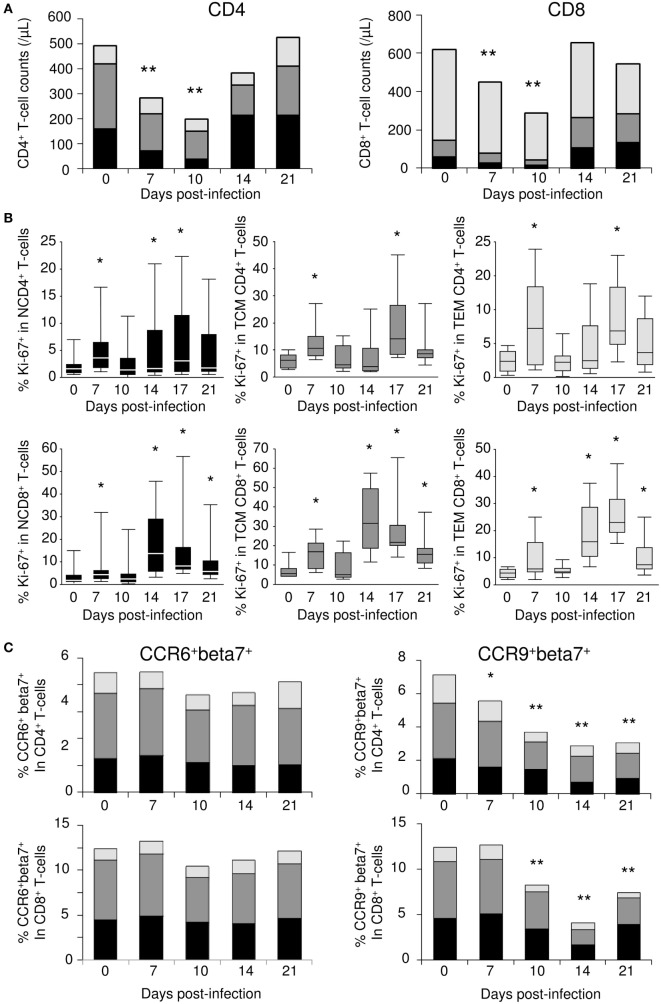
**Changes in blood T-cell subsets during simian immunodeficiency virus (SIV) infection**. **(A)** Changes in the numbers of naïve T-cells (N—CD95^−^CD28^Low^; black), central memory (TCM—CD95^+^CD28^Hi^; dark gray), and effector memory (TEM—CD95^+^CD28^−^; light gray) T-cell counts; CD4^+^ (left panel) and CD8^+^ (right panel) T-cell subsets were evaluated by flow cytometric analysis in macaques (*n* = 10) longitudinally sampled over the first 3 weeks of SIV infection. **(B)** Ki-67 expression in naïve (left panels), TCM (central panels), and TEM (right panels) subsets of CD4^+^ (top panels) and CD8^+^ (bottom panels) T-cells, assessed by flow cytometry. **(C)** The frequency of CCR6^+^beta7^+^ (left panels) and CCR9^+^beta7^+^ (right panels) within CD4^+^ (top panels) and CD8^+^ (bottom panels) naïve (black), central memory (dark gray), and effector memory (light gray) T-cells were evaluated by flow cytometric analysis in macaques (*n* = 6) sampled longitudinally during the first 3 weeks of SIV infection. Statistically significant differences between pre- and post-infection samples are shown (Wilcoxon signed-rank tests; *p < 0.05, **p < 0.01).

Given the presence of important T-cell attracting chemokines in the small intestine of acutely SIV-infected macaques, we investigated whether cell migrations could be observed from the blood. Blood samples were collected over the first 21 days of SIV infection (*n* = 10). Severe depletion of both CD4^+^ and CD8^+^ T-cells characterized the first 10 days of infection (Figure 7A). Naïve T-cells were the most affected subsets during the early phase of SIV infection, with decreases of 5.8- and 4.8-fold at day 10 in CD4^+^ and CD8^+^ T-cell subsets, respectively. By contrast, effector memory T-cell counts barely changed, while a twofold to threefold reduction was observed for central memory T-cells. Following this initial contraction phase, peripheral T-cell counts rebounded by day 14 pi. At this time point, most of the increases in the CD4^+^ T-cell compartment occurred within the naïve subset, while all CD8^+^ T-cell subset numbers increased (Figure 7A). When we examined Ki-67 proliferation marker within circulating T-cells, we identified a significant transient increase in the frequency of Ki-67^+^ cells at day 7 in all T-cell compartments (Figure 7B). However, given the observed T-cell count decreases, these increased frequencies did not translate into any significant change in the numbers of circulating Ki-67^+^ cells. By day 14 pi, a large proportion of the cells that returned to the peripheral blood expressed Ki-67 antigen. This second wave of Ki-67 expression was observed at day 14 pi in naïve CD4^+^ and CD8^+^ T-cells, as well as in memory CD8^+^ T-cells (Figure 7B). Finally, memory CD4^+^ T-cell cycling was detectable at day 17 pi (Figure 7B).

In the same samples, we evaluated cell surface expression of CCR6 (CCL20 receptor) and CCR9 (CCL25 receptor), both of which are involved in intestinal T-cell homing. We also assessed the cell surface expression of β7 integrin, which, in association with α4 integrin, acts as a specific docking molecule to gut endothelial cells. While the frequency of CCR6^+^β7^+^ cells remained constant in all T-cell subsets, we observed a gradual decrease in the frequency of CCR9^+^β7^+^ cells throughout the follow-up period, leading to a twofold to fourfold reduction at day 14 (Figure 7C). Similarly, a twofold reduction in the frequency of CCR9^+^ cells within β7^+^ CD4^+^ and CD8^+^ T-cells was observed at day 14 (data not shown). Together with CCL25 overexpression in the small bowel, these data suggest that circulating immune cells presenting ileal imprinting preferentially migrated into the small intestine during the first 2 weeks of acute SIV infection.

Altogether, these data suggest that chemokine expression in organs, and in particular in the small intestine, triggers massive recruitment of circulating T-cells from peripheral blood. By day 14, cells migrating back into the circulation express the Ki-67 proliferation marker, suggesting that these cells have undergone either antigen priming or cytokine (IL-7) stimulation, especially within the small intestine.

## Discussion

The gastrointestinal tract is one of the first targets of HIV/SIV infection; this results in major changes to the proportions of immune cell subsets within the LP (42). We here evidenced an over production of IL-7 during the first days of acute SIV infection, especially within the small intestine, which may participate to the characteristic cytokine storm occurring during the acute phase of SIV/HIV infection (21). Interestingly, similar enhancements of local IL-7 expression were recently observed in mice infected with *C. rodentium*, and in humans infected with *Helicobacter pylori* (23, 43). Earlier and stronger viral infection in the ileum compared to LNs or to other parts of the gut suggests that the presence of infected cells directly triggers local IL-7 production. Since homeostatic IL-7 expression in the gut is mostly restricted to epithelial cells (9), it is possible that these cells, which are often the first targets of enteric pathogens, could also be producing IL-7 in response to infection, as evidenced in the *C. rodentium*-infected colon (23). However, HIV and SIV do not infect epithelial cells. It is possible, therefore, that IL-7 expression by mucosal cells could also be a consequence of inflammatory cytokine expression by the infected cells; the mucosally produced IL-7 could subsequently act on epithelial cells or other cell types in the mucosa (myeloid cells, endothelial cells, fibroblasts, etc.). Indeed, IFN-γ stimulates IL-7 expression by intestinal epithelial cell line (44). By contrast, IL-1β inhibits IL-7 production but stimulates IL-7Rα, leading to sensitization of the epithelial cells to IL-7 stimulation (44). Similar stimulation cascade was demonstrated in the mouse salivary glands and lungs where epithelial cells express IL-7 after poly I:C or IFNs (α and γ) stimulation, leading to local specific chemokine expressions (45, 46).

Aside from its major role in peripheral T-cell homeostasis, we demonstrated that systemic administration of IL-7 to healthy macaques triggers T-cell homing into organs (37). In the ileum, such migration might be a consequence of the expression of CCL25, which was increased as early as 1 day following IL-7 treatment (37). However, we here evidenced similar overexpression of CCL2, CCL3, CCL4, CCL28, and CXCL8 in the ileum of IL-7-treated macaques, chemokines that can also, among other cell subsets, trigger T-cell homing. Interestingly, CCL4, CCL25, and CCL28 expressions directly correlate with IL-7 expression in the infected ileum, suggesting that during acute SIV infection, IL-7 triggered the expression of these chemokines within the ileum. However, it cannot be excluded that other stimuli also participated in the induction of chemokine production and immune cell homing within the inflamed mucosa. These stimuli could include infected cells, virions, and/or the production of other virally induced factors such as the inflammatory cytokines that are produced in the early phase of immune responses. Indeed, CCL2, CCL3, CCL19, and CXCL10 expression correlated with the viral load within the ileum mucosa of SIV-infected macaques. Altogether, our observations are consistent with IL-7 being an infection-induced early danger signal that may participate in driving the massive changes that occur within the small intestine chemokine network in response to infection.

To assess the consequences of alterations to the intestinal chemokine network on T-cell homeostasis, we developed an imaging method that allowed for the reliable cell quantification of large mucosal surfaces (25 mm^2^). In keeping with recent observations in acutely HIV-infected humans (34, 47), absolute CD4^+^ T-cell counts remained stable in the gut mucosa during the first 2 weeks of SIV infection. By contrast, we showed that circulating CD4^+^ and CD8^+^ T-cell counts decreased rapidly in SIV-infected Chinese rhesus macaques, consistent with our previous observations (48). These results suggest that CD4^+^ and CD8^+^ T-cells homed into tissues. Indeed, in the presence of replicating virus that could cause CD4^+^ T-cell death, the observed stability of CD4^+^ T-cell counts in the ileal LP suggests either increased cell homing to the mucosa or inhibited egress from the gut. By contrast, increased CD8^+^ T-cell counts in the LP strongly suggest both increased homing and local proliferation in response to antigenic stimulation. Together with preserved CD4^+^ T-cell counts, increased CD8^+^ T-cell counts in mucosal tissues results in reduced mucosal CD4^+^ T-cell percentages as reported in acutely infected macaques (49). In the infected ileal mucosa, T-cell homing could be triggered by CCL19, whose expression was increased at day 10. Indeed, in macaques, the majority of circulating T-cells express CCR7, the CCL19 receptor (37). Moreover, *in vivo*, IL-7-stimulated T-cells overexpress CCR9 and CXCR4 (37), as well as the integrin α4β7 (38). In SIV-infected macaques, the strong depletion of circulating β7^+^CCR9^+^ cells we observed is consistent with the homing of these cells into CCL25-expressing intestinal mucosa. Thus, induced expression of both CCL19 and CCL25, as well as constitutive expression of CXCL12 in the infected ileum may participate in the specific homing of most T-cell subsets. Whether T-cell gut homing is beneficial to the host or feeds the virus remains to be fully established. While CCR7 and CCR9-driven homing into the small intestine associated with local CD4^+^ T-cell depletion during acute SIV infection in Indian rhesus macaques (50), it should be noticed that Chinese rhesus macaques usually demonstrate a slower disease progression rate than macaques of Indian origin (51, 52). Homing of CD4^+^ T-cells to the gut during the acute phase of infection in this model may, as in humans (34), trigger longer preservation of gut-associated CD4^+^ T-cell compartment, leading to delayed pathogenesis. However, mobilization of α4β7 CD4^+^ T-cells to the gut of acutely infected Indian macaques was shown to participate to the initial development of SIV replication leading, in this highly pathogenic model, to gut damage (53, 54).

Coincident with their plateauing in the LP, circulating CD8^+^ T-cell counts rebounded by day 14; a large proportion of these cells expressed the Ki-67 proliferation marker. A similar expansion of activated CD8^+^ T-cells was observed in acutely HIV-infected patients a week after the peak of viremia, principally due to the expansion of anti-HIV CTL clones (55). It is thus probable that among the cells that initially homed in response to local chemokine expression in the infected tissues, SIV-specific CD8^+^ T-cells proliferate in response to SIV antigens, and then recirculate by day 14. This recirculation is concomitant with the drop in intestinal chemokine transcription. It is likely that stimulation by locally produced IL-7 plays a role in this proliferation. Similarly, IL-7R internalization triggered by IL-7 stimulation may explain the reduced CD127 expression characterizing circulating HIV-specific CTLs during hyperacute infection in patients (55). In the CD4^+^ T-cell compartment, the egress of Ki-67^+^ cells was delayed compared to the CD8^+^ compartment. This observation might be the result of the slower expansion of SIV-specific CD4^+^ T-cells as a consequence of their preferential targeting during acute infection, similar to what was reported in HIV individuals (56). In both CD4^+^ and CD8^+^ compartments, naïve T-cells also overexpressed Ki-67 at day 14 pi.

In uninfected macaques, the LP contained equal amounts of CD3^+^CD4^+^ T-cells and CD3^−^CD4^+^ APCs. By contrast, the latter subset represented the vast majority of CD4^+^ cells in the submucosa and was largely composed of PM-2K^+^, CD68^+^, CD169^+^, and DC-SIGN^+^ macrophages that did not proliferate in the tissues. DC-SIGN^+^ macrophages were recently observed in the colonic LP (57). In mice, this population is constantly renewed by circulating monocytes and participates in the surveillance of mucosal integrity through the chemokine-dependent recruitment of inflammatory monocytes (58, 59). In our study, we observed that throughout the first days of SIV infection, CD4^+^ macrophages almost completely disappeared from the submucosa, concomitantly with their increase in the LP. Subsequently, a significant number of ileal DC-SIGN^+^ macrophages appeared to leave the intestinal tissue based on the observation that by day 14, the size of this population was approximately half reduced compared to the size of the initial population. Similar macrophage behavior has been observed in the penile epithelium following HIV infection in explant cultures (60). It is conceivable that the increased LP macrophage content could be a consequence of enhanced trafficking of blood monocytes into intestinal tissues as has been observed in the chronic phase of HIV infection. Indeed, an accumulation of macrophages in the LP of untreated HIV-infected patients has been reported as a response to local infection (61). Alternatively, chemokine gradients within the intestinal mucosa may have induced submucosal macrophages migration to the LP, positioning the cells closer to the epithelium, the usual portal of entry for gut pathogens. It is possible that these macrophages could also transport SIV/HIV particles (bound to DC-SIGN molecules) and participate to establishment of viral reservoirs.

Among the chemokines involved in macrophage homing (62, 63), CCL3, CCL4, and CCL5 were overexpressed in the ileum of SIV-infected macaques. Similarly, intestinal CCL5 production was also demonstrated following enterovirus infection (64). However, in the SIV-infected ileum, macrophage recruitment preceded CCL5 chemokine overexpression. Nevertheless, some chemokine gradients (i.e., CCL2, CCL3, CCL4, CCL5, CCL20, and CXCL8) are not only driven by *de novo* synthesis but also by rapid release of intracellular chemokine stocks upon cell activation (65–69). It is thus possible that the recruitment of monocytes/macrophages into/within the mucosa starts in response to exocytosed chemokines before transcription increases. In addition, activated macrophages secrete CCL2, CCL3, CCL5, CXCL8, and CXCL10 and can thus establish a positive feedback loop to attract new monocytes/macrophages into the ileum (70, 71).

Finally, one can expect CCL3, CCL4, CCL5, and CXCL10 to also stimulate NK cell migration to the infected ileum. Indeed, in the ileal LP of infected macaques, we observed a 2.4-fold increase in CD3^−^CD8^+^ cell counts (data not shown), suggesting NK cell infiltration.

Altogether, our observations suggest that IL-7 is one of the key participants in the early phase of SIV infection. Triggering chemokine expression within the ileum, it is an early signal that impact both innate (monocytes/macrophages/NK) and adaptive (T-cells) antiviral immune responses. Moreover, CD8^+^ T-cell and NK cell recruitment into the gut and activation of anti-HIV CTLs may limit viral expansion to blunt the peak viral load; unfortunately, these efficient immune mechanisms are hijacked by HIV/SIV. Indeed, IL-7-induced alterations in chemokine networks during acute HIV/SIV infection and the recruitment of CD4^+^ T-cells to the sites of viral replication, together with the increased presence of DC-SIGN^+^ macrophages, may participate in viral spread and in the establishment of viral reservoirs. As a consequence, it is important to investigate to what extent intervening with cell trafficking into the intestine, in conjunction with early antiviral therapy, could help protect the gut and limit the establishment of HIV reservoirs.

## Ethics Statement

All the animals included in this study were housed, cared for, and handled in BSL2 (uninfected macaques) or BSL3 (SIV-infected macaques) facilities at the Institut Pasteur (Paris, France; accreditation no. A 78-100-3) and IDMIT (“Infectious Disease Models and Innovative Therapies” at the CEA “Commissariat à l’Energie Atomique,” Fontenay-aux-Roses, France; accreditation no. C 92-032-02) NHP facilities, licensed by the French Ministry of Agriculture, in accordance with European guidelines for animal care (European directive 86/609, “Journal Officiel des Communautés Européennes,” L358, December 18, 1986). The CEA is in compliance with Standards for Human Care and Use of Laboratory of the Office for Laboratory Animal Welfare (OLAW, USA) under OLAW Assurance number #A5826-01. This study and the procedures used were approved by the “Comité Régional d’Ethique en Expérimentation Animale Ile-de-France—Paris—Comité 1,” which follows the governance of the “Charte nationale portant sur l’expérimentation animale” according to the EU Directive 2010/63 on the protection of animals used for scientific purposes (notifications no. 2011-0001 and 2012-0003). All experimental procedures were carried out in strict compliance with European guidelines for NHP care (European Directive 86/609 then; as of January 2013, EU Directive N 2010/63/EU) for protection of animals used for experimentation and other scientific purposes and the Weatherall report. Food and water were provided *ad libitum*. Temperature was maintained constant (22 ± 2°C). Air was renewed at least 20 times per hour. Fluorescent light was provided with a 12:12 h light–dark cycle. All efforts were made to minimize suffering, including improving housing conditions and providing enrichment opportunities (e.g., provision of monkey biscuits supplemented with fresh fruit and constant water access, objects to manipulate, interaction with caregivers and research staff). Animals were kept in cages in compliance with European regulations in terms of floor surface per animal. All manipulations (injections, blood draws, and euthanasia) were performed under anesthesia (tiletamine/zolasepam; 4.6 mg/kg). Euthanasia was performed by intravenous injection of pentobarbital (10 mL). Permanent veterinarians managed the animal facilities and were responsible for the well-being of the experimental animals. The animal facilities were run by teams of animal caretakers under the supervision of animal technicians. According to European and French regulations, animal caretakers have received a specific training in laboratory animal science. Technicians who were doing experiments on live animals had received the appropriate training. Biohazards were handled in BSL2 and BSL3 facilities according to French legislation.

## Author Contributions

Conceptualization: RC and AC-C. Methodology: RP, MR, RC, and AC-C. Investigation: RP, MR, AC-C, SF-M, VF-M, JD, and BC-d-M. Formal analysis: RC and AC-C. Software: TG. Writing—original draft: RC and AC-C. Writing—review and editing: RP, MR, J-PR, RC, and AC-C. Funding acquisition: RC and AC-C. Supervision: RC and AC-C.

## Conflict of Interest Statement

The authors declare that the research was conducted in the absence of any commercial or financial relationships that could be construed as a potential conflict of interest. The handling editor declared a shared affiliation, though no other collaboration, with the authors and states that the process nevertheless met the standards of a fair and objective review.

## Footnotes

^1^http://imagej.nih.gov/ij.

^2^http://www.real-statistics.com.
