# Supplementary material for: Acute Simian Immunodeficiency Virus Infection Triggers Early and Transient Interleukin-7 Production in the Gut, Leading to Enhanced Local Chemokine Expression and Intestinal Immune Cell Homing
Source: Front Immunol. 2017 May 19;8:588. doi: 10.3389/fimmu.2017.00588 (PMC5437214; doi:10.3389/fimmu.2017.00588)
Supplement: Supplementary file 1 [file data_sheet_1.pdf]

Supplementary Material

Acute SIV infection triggers early and transient IL-7 production in the gut leading to enhanced local chemokine expression and intestinal immune cell homing

Rosalie PONTE[1,2,3], Magali RANCEZ[1,2,3,#], Suzanne FIGUEIREDO-MORGADO[1,2,3,#], Jacques DUTRIEUX[1,2,3], Véronique FABRE-MERSSEMAN[1,2,3], Bénédicte CHARMETEAU-DE MUYLDER[1,2,3], Thomas GUILBERT[1,2,3], Jean-Pierre ROUTY[4], Rémi CHEYNIER[1,2,3,*] and Anne COUËDEL-COURTEILLE[1,2,3,5]

*** Correspondence:** Rémi Cheynier, email: remi.cheynier@inserm.fr

1 Supplementary Figures and Tables

1.1 Supplementary Figures

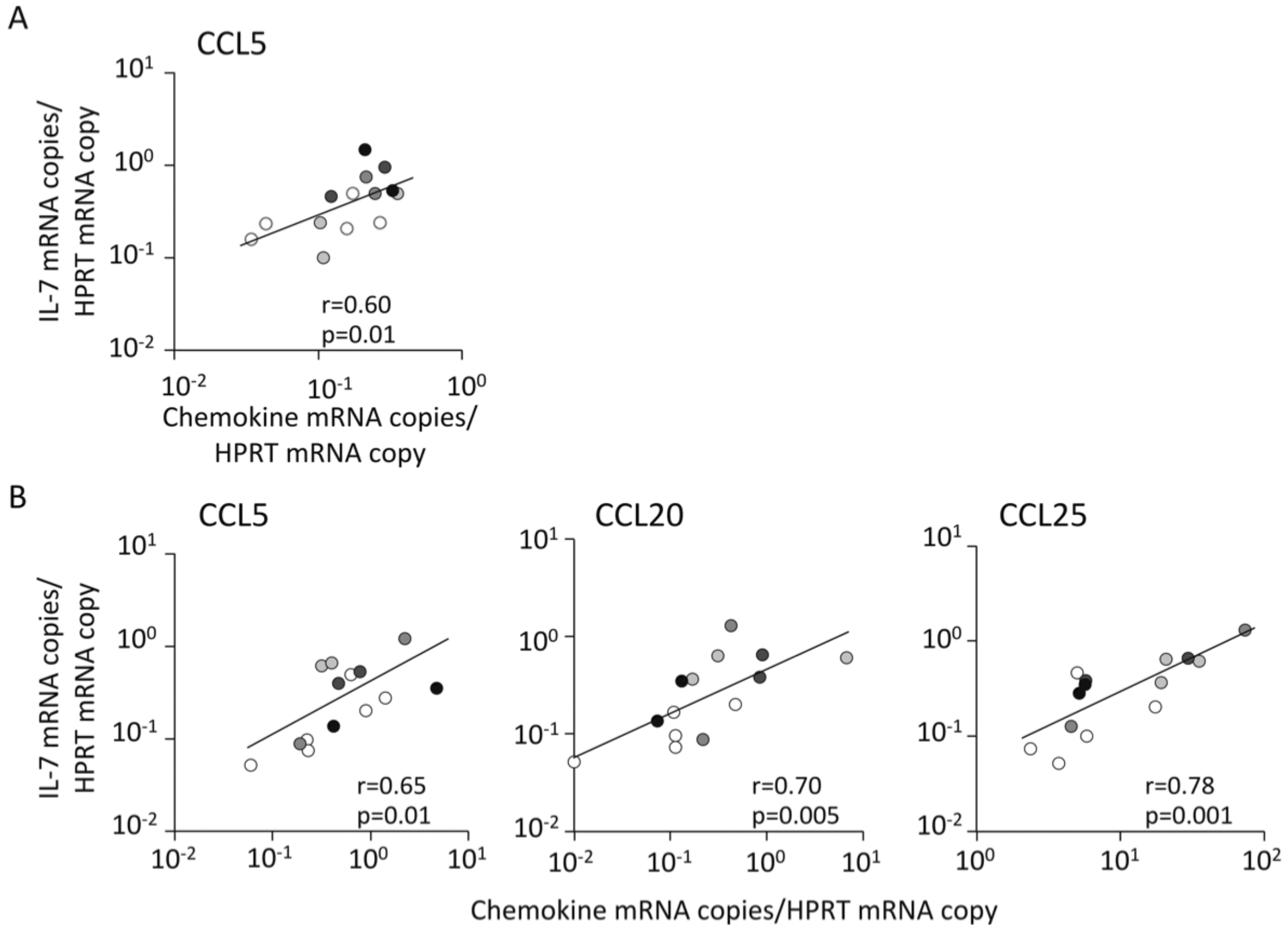

Supplementary Figure 1. Correlations between IL-7 and chemokine mRNA expressions in the duodenum (A) and the jejunum (B) of SIV-infected macaques (0 to 10 days post-SIV infection).

Each point represents one macaque. White circles represent uninfected controls; light grey, medium grey, dark grey and black circles represent infected macaques sampled at day 3, 7, 10 or 14, respectively. The regression lines, the Spearman's rank correlation value and the associated probability are shown for each chemokine.

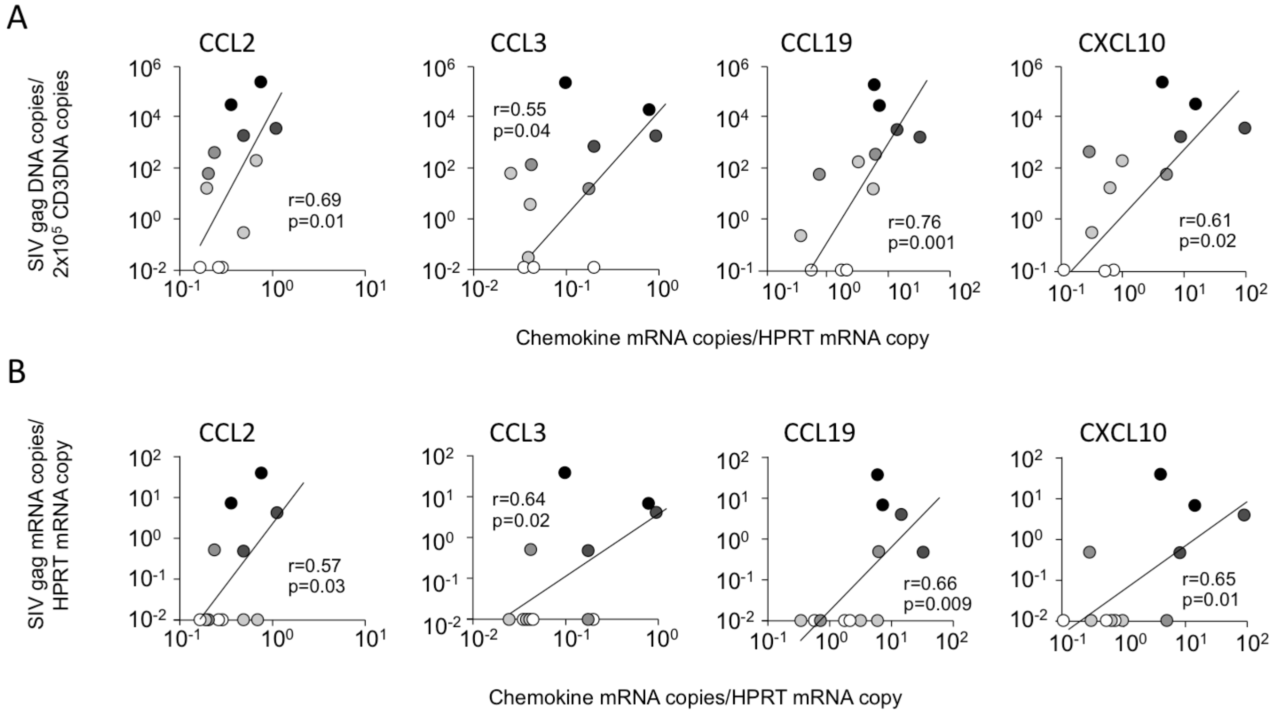

Supplementary Figure 2. Correlation between viral load and chemokine expression in the ileum of SIV-infected rhesus macaques.

SIV DNA load (SIV gag DNA/10^5cells), Viral load (SIV gag mRNA copies/HPRT mRNA copy) and CCL2, CCL3, CCL19, CXCL10 chemokine expression (chemokine mRNA copies/HPRT mRNA copy) were quantified in the ileum of SIV-infected macaques (day 3 to day 10 pi). (A) correlations between SIV DNA and chemokine mRNA, (B) correlations between SIV RNA and chemokine mRNA. Each point represents one individual sample. Light grey, medium grey, dark grey and black circles represent infected macaques sampled at day 3, 7, 10 or 14, respectively. The regression lines, the Spearman's rank correlation value and the associated probability are shown for each chemokine.

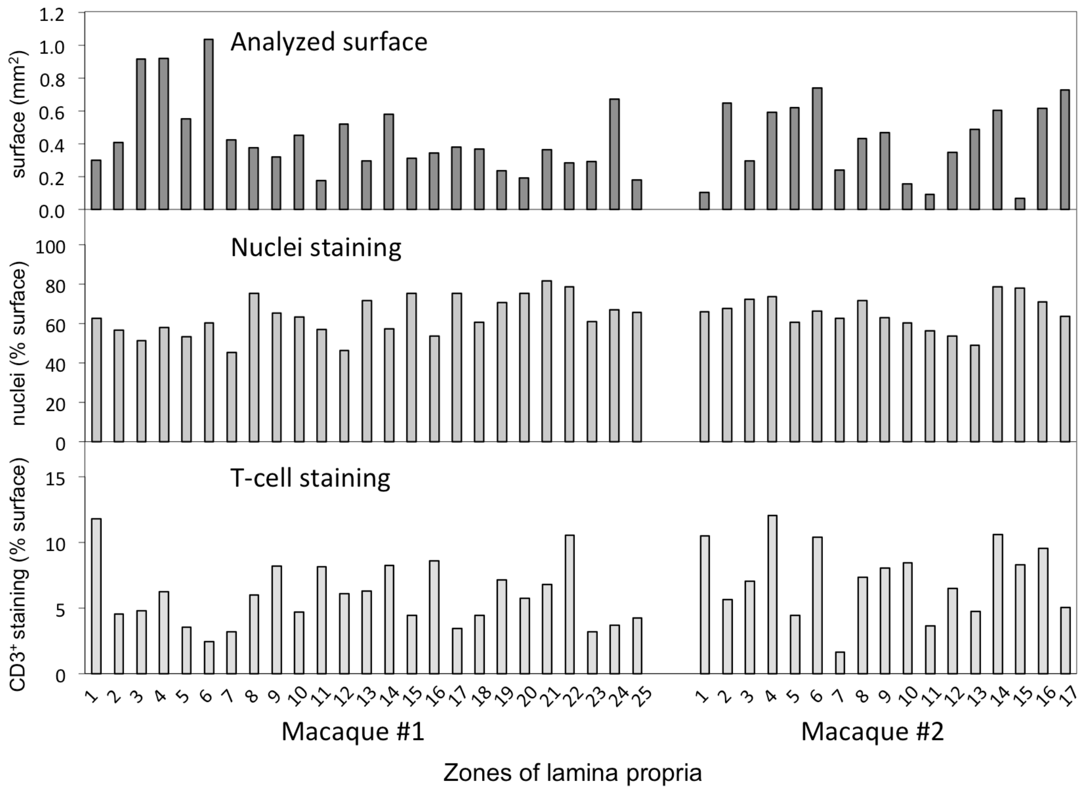

Supplementary Figure 3. CD3 staining in the ileum of uninfected rhesus macaques.
Ileal tissues sampled from 2 healthy macaques were labeled with DAPI (nuclei) and anti-CD3 antibody. Twenty-five and 17 different zones of the LP were defined for macaques 1 and 2, respectively. For each zone, tissue surface was measured (top panel). The proportions of LP surfaces occupied by nuclei (central panel) and by CD3$^+$ T-cells (bottom panel) were calculated for each individually analyzed zone.

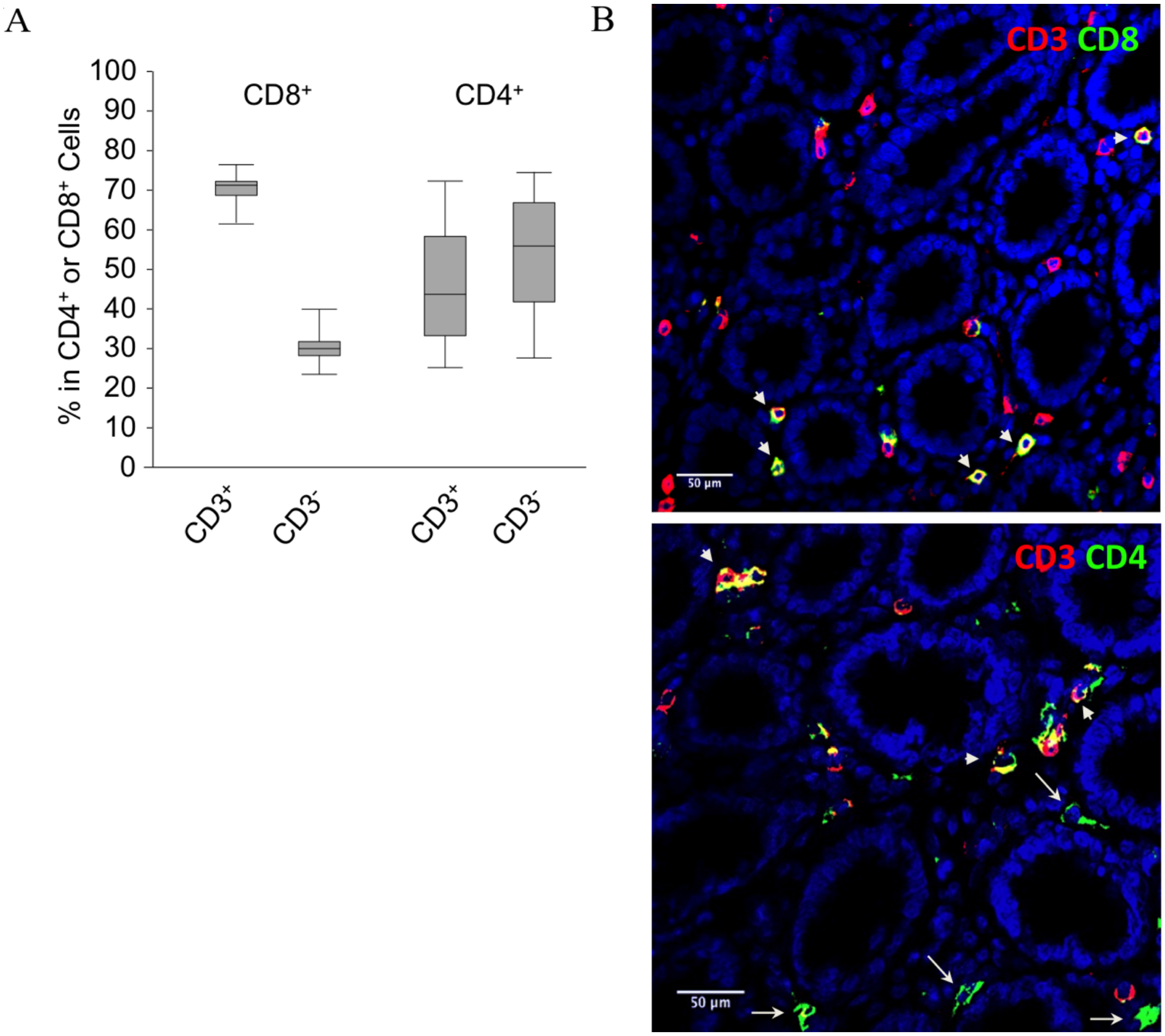

Supplementary Figure 4. Quantifying non-T-cells in the LP of healthy rhesus macaques.

Ileal tissues sampled from 2 healthy macaques were co-labeled with anti-CD3 antibody and with either anti-CD8 or anti-CD4 monoclonal antibodies. (A) The percentages of CD8$^+$ T-cells (CD3$^+$CD8$^+$) and CD8$^+$ non-T-cells (CD3$^-$CD8$^+$) within CD8$^+$ surfaces, as well as CD4$^+$ T-cells (CD3$^+$CD4$^+$) and CD4$^+$ non-T-cells (CD3$^-$CD4$^+$) within CD4$^+$ surfaces, were calculated for each individual LP area. (B) Representative examples of LP staining with anti-CD3 (red) and anti-CD8 (green) (top panel) and anti-CD3 (red) and anti-CD4 (green) (bottom panel). Arrows identify CD4$^+$ non-T-cells and arrowheads indicate either CD8$^+$ (top panel) or CD4$^+$ (bottom panel) T-cells.

1.2 Supplementary tables

Supplementary Table 1. Oligonucleotides used to amplify IL-7, chemokines and HPRT

Name	Sequence	Name	Sequence
HPRT-Out5	CTGAACGTCTTGCTCGAGAT	HPRT-In5	CACATTGTAGCCCTCTGTGT
HPRT-Out3	CGACCTTGACCATCTTTGGA	HPRT-In3	CTGACCAAGGAAAGCAAAGT
CCL2-Out5	AACATCCAGTGCTCAAACTGAA	CCL2-In5	CTGCTCATAGCAGCCACCTTCA
CCL2-Out3	TCCAGGTGGTCCATGGAAT	CCL2-In3	TCCTGAACCCACTTCTGCTT
CCL3-Out5	CATTCCATCACCTGCTCAGAA	CCL3-In5	CAACCGGATCTCAGCAACAT
CCL3-Out3	TCCAGGTCGCTGACGTATTT	CCL3-Int3	GCCGGCCTCTCTTGGTTA
CCL4-Out5	CCATGAAGCTGCGCGTGA	CCL4-In5	CCCACCTCCTGCTGCTT
CCL4-Out3	CTCAGTTCAGTTCCAGGTCA	CCL4-In3	GCAGACTTGCTTCCCTCTTTT
CCL5-Out5	GCTGTCATCCTCGTTGCTA	CCL5-In5	ACATGCCTCAGACACCACA
CCL5-Out3	CTCATCTCCAAAGAGTTGATGTA	CCL5-In3	TACTCCCGAACCCATTTCTT
CCL11-Out5	TGAAGGTCTCCACAACACT	CCL11-In5	TGCAACCACCTGCTGCTTTA
CCL11-Out3	TGGCTTTGGAGTTGGAGAT	CCL11-In3	TTGGAGATTTTCGGTCCAGATA
CCL19-Out5	ACCGTTGGCCTGCCTCTGTT	CCL19-In5	CAGCCTGCTGGTTCTCTGGA
CCL19-Out3	CTGCTGCGGCGCTTCATCTT	CCL19-In3	TCCTCTGCAGTCTCTGGAT
CCL20-Out5	CCATGTGCTGTACCAAGAGT	CCL20-In5	TTGCTCCTGGCTGCTTTGA
CCL20-Out3	TGCTGAGGCGACGTACAATA	CCL20-In3	CCCAGGTCTGCTTTGGATT
CCL21-Out5	CCTCAGCTCTGGCCTCTTA	CCL21-In5	ATGGCTCAGTCACTGGCTCT
CCL21-Out3	TCACTGGGCTATGGCCCTTTA	CCL21-In3	CTATGGCCCTTTAGGGGTCT
CCL25-Out5	CACACCCAAGGTGTCTTTGA	CCL25-In5	TTATCGGATCCAGGAGGTGA
CCL25-Out3	TTAGCTGATGTCAGGAGGGA	CCL25-In3	CTGCTGGTGGGATTGCTAAA
CCL28-Out5	AAGGCTCCTGGAAAGAGTGAA	CCL28-In5	TCGCATCCAGAGAGCTGAT
CCL28-Out3	GTTTCGTGTTTCCCCTGATGT	CCL28-In3	CTGATGTGCCCTGTTACTGT
CXCL8-Out5	GAACCATCTCGCTCTGTGTA	CXCL8-In5	ATCTCGCTCTGTGTAAACATGA
CXCL8-Out3	GCTCTCTTCCATCGGAAAGT	CXCL8-In3	CTGTATTGGCACAGTGTGGT
CXCL10-Out5	TGATTTGCTGCCTTGTCTTTCT	CXCL10-In5	CTCTCTCAAGAACTGTACGCT
CXCL10-Out3	ACCTCTTCTCACCCTTCTTTT	CXCL10-In3	CATGTGGACAAAATTGACTTGGA
CXCL12-Out5	GTCAGCCTGAGCTACAGAT	CXCL12-In5	TACAGATGCCCATGCCGATT
CXCL12-Out3	TTTCTCCAGGTACTCCTGAA	CXCL12-In3	TCGGGTCAATGCACACTTGT
IL-7-Out5	TTTGGACTTCCTCCCCTGAT	IL-7-In5	CCCCTGATCCTTGTTCTGTT
IL-7-Out3	GTGTTCTTTAGTGCCCATCAAAAT	IL-7-In3	GCGAGCAGCACGGAATAAAA

Supplementary Table 2. Oligonucleotides used to amplify SIV gag and CD3γ house keeping gene

Name	Sequence	Name	Sequence
CD3-Out5	ACTGACATGGAACAGGGGAA	CD3-In5	GGCTATCATTCTTCTTCAAGGTA
CD3-Out3	AGCTCTGAAGTAGGGAACATAT	CD3-In3	TTCCTGGCCTATGCCCTTTT
Gag-SIV-Out5	CAACAAGGACAGCTTAGGGA	Gag-SIV-In5	CCGTCAGGATCAGATATTGCA
Gag SIV-Out3	TTGACAGGCCGTCAGCATTT	Gag SIV-In3	GAAACTATGCCAAAAACAAGT

Supplementary Table 3. Antibodies used for polychromatic flow cytometry analysis

Antibodies

**Target epitope**	**Fluorochrome-conjugated**	**Clone**	**Supplier**	**Supplier details**
CD3	PE-Cy7 *(phycoerythrin (PE)-Cy7)*	SP34-2	BD Biosciences	Le Pont de Claix, France
CD4	PerCP-Cy5.5 *(peridinin chlorophyll protein-Cy5.5)*	L200	BD Biosciences	Le Pont de Claix, France
CD8	PB *(pacific blue)*	RPA-T8	BD Biosciences	Le Pont de Claix, France
CD28	PE *(phycoerythrin)*	CD28.2	BD Biosciences	Le Pont de Claix, France
CD95	APC *(allophycocyanin)*	DX2	BD Biosciences	Le Pont de Claix, France
CD127	Biotin + Streptavidin-phycoerythrin (PE)-Texas Red	MB15-18C9	Miltenyi	Paris, France
Ki-67	FITC *(fluorescein isothiocyanate)*	MIB-1	DAKO	Les Ulis, France
β7	Biotin + Streptavidin-PO (*pacific orange*)	FIB504	BioLegend	St-Quentin en Yvelines, France
CCR6	FITC *(fluorescein isothiocyanate)*	53130	R&D systems	Lille, France
CCR9	FITC *(fluorescein isothiocyanate)*	112509	R&D systems	Lille, France

Supplementary Table 4. Antibodies used for the immunostaining of ileal sections

Primary antibodies

Target epitope or cell type	Isotype	Clone	Supplier	Supplier details	Concentration (µg/ml)
CD3	Rabbit IgG	(polyclonal)	DAKO	Trappes, France	2.5
CD4	Mouse IgG1	L200	BD Biosciences	Le Pont de Claix, France	5
CD4	Mouse IgG2b	OKT4	BioLegend	St-Quentin en Yvelines, France	10
CD8	Mouse IgG1	RPA-T8	BD Biosciences	Le Pont de Claix, France	5
HLA-DR	Mouse IgG2b	TU36	BD Biosciences	St-Quentin en Yvelines, France	0.1
Macrophages	Mouse IgG1	PM-2K	AbD Serotec	Düsseldorf, Germany	0.5
CD68	Mouse IgG1	KP1	DAKO	Trappes, France	4
DC-SIGN	Mouse IgG2b	120507	R&D systems	Lille, France	5
CD11c	Mouse IgG1	3.9	BioLegend	St-Quentin en Yvelines, France	5
CD169	Mouse IgG1	HSn 7D2	Abcam	Paris, France	5.5
CD86	Mouse IgG2b	IT2.2	BD Biosciences	Le Pont de Claix, France	2
CD83	Mouse IgG2b	HB15A	Beckman Coulter	Marseille, France	10
Ki-67	Rabbit IgG	(polyclonal)	Merck Millipore	Fontenay sous Bois,	Not specified
IL-7	Rabbit IgG	(polyclonal)	Abcam	France	4.3
CK18	Guinea-pig	(polyclonal)	Abcam	Paris, France	Not specified
Ezrin	Rabbit	(polyclonal)	Gift from P. Mangeat	Paris, France CNRS, Montpelier, France	Not specified

Secondary antibodies

Target species	Host species	Conjugate	Supplier	Supplier details	Concentration (µg/ml)
Mouse (IgG1, IgG2a or IgG2b)	Goat	Alexa Fluor® 546 or 488	Molecular Probes	Cergy Pontoise, France	5
Rabbit	Goat	Alexa Fluor® 546 or 488	Molecular Probes	Cergy Pontoise, France	5
Guinea pig	Goat	Alexa Fluor® 488	Thermo Fisher	Villebon, France	5

Supplementary Table 5. Quantification of SIV RNA in tissues sampled from SIV-infected rhesus macaques.

SIV RNA	Mac #	Axillary LN	Mesenteric LN	Duodenum	Jejunum	Ileum	Asc. Colon	Desc. Colon	Rectum
Day3	6	- [a]	-	-	-	-	-	-	-
	7	-	-	-	-	-	-	+++	-
	8	+	-	-	-	-	-	-	-
Day7	9	-	+	-	+	-	++	-	-
	10	-	-	-	-	-	-	-	-
Day10	11	+	+++	+++	-	++	+	++	++
	12	++	+++	++	-	+++	+	+	++
Day14	13	++++	++++	++++	++++	++++	++++	++++	++++
	14	+++	+++	+++	++++	+++	++	+++	+++

[a] - : Undetectable; +: 0 < X ≤ 0.1 copy/HPRT copy; ++: 0.1 < X ≤ 1 copy/HPRT copy; +++: 1 < X ≤10 copies/HPRT copy; ++++: X > 10 copies/HPRT copy.
